# Homelessness and self-rated health: evidence from a national survey of homeless people in Spain

**DOI:** 10.1186/s12889-019-7380-2

**Published:** 2019-08-09

**Authors:** Fernando Fajardo-Bullón, Igor Esnaola, Isobel Anderson, Lars Benjaminsen

**Affiliations:** 1 Department of Development and Educational Psychology. Faculty of Education, University of Extremadura, Badajoz, Spain; 2 Department of Development and Educational Psychology. Faculty of Education (Avenida de Tolosa, 20018, San Sebastián), University of the Basque Country, UPV/EHU, Leioa, Spain; 3 Faculty of Social Sciences, University of Stirling, Stirling, UK; 4 The Danish Center for Social Science Research, København, Denmark

**Keywords:** Spain, Homelessness, Self-rated health, Drug consumption, Descriptive survey study

## Abstract

**Background:**

Internationally, acute homelessness is commonly associated with complex health and social care needs. While homelessness can be understood as an outcome of structural housing exclusion requiring housing led solutions, the health care issues faced by homeless people equally require attention. A substantive evidence base on the health needs of homeless people exists, but relatively little is known about what influences the self-rated health of homeless people. This article presents new evidence on whether drug use (alcohol consumption, ever having used drugs), health variables (visiting a hospital once in the last year, visiting the doctor in the last month, having a health card, sleeping difficulties, and having a disabling impairment) and sociodemographic characteristics are significantly associated with Self-Rated Health (SRH) among Spanish homeless people.

**Method:**

The approach applies secondary analysis to cross-sectional data from a sample of 2437 homeless adults in Spain (83.8% were male). Multinomial logistic regression modelling was used to analyse the relationships between drug use, other health variables and SRH.

**Results:**

Being male, an abstainer, having a health card and being in the youngest age groups were significant factors associated with perceived good health. On the other hand, ever having used drugs, having been a night in hospital, having gone to the doctor in the last month, having sleeping difficulties, having a disabling impairment and being in the older age group were all significant risk factors associated with perceived poor health.

**Conclusions:**

These results help to improve understanding of the key factors that influence the SRH among homeless people. The findings can contribute to development and delivery of preventive policies, suggesting that interventions to reduce drug consumption and ensure access to a health card/health services, as well as enhancing services for older, female and disabled homeless people are all measures which could improve health and well-being for those who face homelessness. Effective housing interventions (e.g. Housing First or Permanent Supported Housing programmes) are equally important to underpin the effectiveness of measures to improve the self-rated health of homeless people.

## Background

In recent years, poverty, inequality situations and social exclusion have increased in Spain, becoming the highest in the European Union [1]. The last Spanish Survey on Centres and Services to Support Homeless People showed an increase of 20.5%, in the average daily number of homeless people who stayed in shelters in Spain, with an increase in occupancy from 81.78% in 2014 to 85.94% in 2016 [2]. One of the main purposes of the “Comprehensive National Homelessness Strategy 2015-2020” is to prevent homelessness, as well as to reduce the number of persons living on the street and foster an overall reduction in the Spanish homeless population [3].

International evidence indicates that homeless people experience poorer physical and mental health compared to general population [4] and the significance of homelessness for health issues is acknowledged by the World Medical Association (WMA) [5]. Homeless people’s risk of dying prematurely is three to four times that of the general population [6]. Much of the excess mortality is explained by risk factors, like alcohol consumption, drug use [7] and physical health problems [8].

Poor health is commonly associated with homelessness, especially long-term homelessness and acute or street homelessness [9]. Factors such as prolonged outdoor exposure combined with substance use and street culture impact negatively on health and life expectancy [9]. Consequently, homelessness also contributes to the burden of acute and chronic illnesses, disrupts continuity of health care services and, impedes access to health care and the quality of interactions with health care providers [10, 11].

Despite their high health care needs, homeless people often lack access to primary or preventive health services. In addition, transportation to and from medical appointments is often impossible because of timing and expense. Health cards, prescriptions and integrated health services are not usually within their grasp, although health and social policy dictates that they are entitled to these benefits. As a consequence, often the only health care homeless people receive is through the emergency department, which is costly and ineffective [12]. UK evidence indicates that homeless people stay in hospital longer than other low-income patients, which impacts on costs of health care provision [13].

A recent international review [14] concluded that evidence on the health of homeless people in Europe was patchy compared to the US research base. This was especially the case for homeless women, and there remained a need for more research on how homelessness impacts on health and access to health care services. Moreover, there is increasing interest among national governments in obtaining robust information on the homeless population in order to plan housing and care interventions [15].

While there is a substantial research literature on the health of homeless people, homelessness itself also requires to be understood as a structural issue of lacking a suitable home [16–18]. Research has shown that poor housing quality is associated with morbidity related to infectious and chronic diseases, injuries, poor nutrition, asthma, neurologic damage, and mental disorders [19]. Additionally, living in crowded conditions can exacerbate poor health and increase the likelihood of infectious diseases, such as tuberculosis.

Efforts to address homelessness since the 1980s have increasingly acknowledged the importance of housing as an important and cost effective solution to homelessness and health care service delivery [20]. Tackling housing exclusion is also consistent with basic human rights [21]. In response, the US, in particular, has experienced a notable policy change from use of temporary shelters to ordinary housing in the community, with required support services (or ‘permanent supportive housing’, PSH) as a preferred solution to homelessness [9, 22, 23]. Waldbrook (2015) [4] found that quality of housing was important for the healthy aging of formerly homeless people. Settled housing also contributed to participants capacity to eat well and prioritise their own health and wellbeing.

In Europe, the adoption of housing-led solutions to homelessness has tended to reflect the historic development of welfare states, and specifically the existence of an affordable social housing sector (e.g. as in the United Kingdom) and until recently have been much less established in the Mediterranean European states [24]. Recently, however, the Housing First approach has been implemented in some Spanish cities, and voluntary adherence to interventions has shown early results demonstrating improvements in health, including recognition of health problems, acceptance of medical visits, follow-up of appointments and medical guidelines for treatment and, perceptible improvements in general health condition [25].

Despite progress in research and policy, homelessness persists, as does the need to address the adverse health circumstances of homeless people. This paper contributes to this agenda by presenting new evidence on how substance use, health and demographic variables are associated with SRH of homeless people in Spain. Drawing on a robust national data set, the analysis shows how drug use and other health indicators are associated with SRH among the Spanish homeless population. The findings contribute to informing policy and practice to improve the health of homeless people and to alleviate homelessness as a national social issue.

### Self-rated health

Although one of the most analysed variables in epidemiology, Self-Rated Health (SRH), also called self-assessed health or self-perceived health, has rarely been used in studies of health and homelessness. The SRH is usually based on asking individuals to evaluate their health status on a four- or five-point scale and is used to analyse the associated risk factors [26]. Several studies have analysed the relationship between SRH and physical health within the general population. In some studies, no association was found between SRH and health factors such as hospital morbidity, occupational accidents and consumption of medicines among the Spanish general population [27]. However, more recent studies in the general Spanish population have demonstrated the relationship between objective health and SRH [28, 29], physical disability [30], functional performance, physical and social activity [31] or mental health [32]. Since the relationship between SRH and mortality has been demonstrated [33], its use in national and international surveys has increased and SRH has been recommended as a useful and convenient tool in national surveys over recent decades [34].

SRH has become a well-established tool to assist in measuring health characteristics and inequalities [26]. The SRH measure is considered to be highly reliable [35] and particularly useful for predicting future health issues as well as measuring current health [36, 37]. Evidence does suggest that SRH is a more effective predictor of mortality among men than women and that differences also exist in relation to some ethnic and socio economic groups [38]. Likewise, Zajacova and Woo (2016) [39] claim that SRH is less reliable among older populations, possibly due to different self-evaluation in later life, necessitating some caution in considering different age groups.

As discussed above, homelessness is also synonymous with poor health and health–related morbidity. Compared with housed individuals, homeless people experience higher prevalence and incidence of medical and psychiatric morbidity and mortality [40], but homelessness also makes it difficult to obtain health care. Notwithstanding the structural housing exclusion which underpins homelessness, the Institute of Medicine summed up the interactions between homelessness and health as: (1) some problems precede and causally contribute to homelessness; (2) others are consequences of homelessness; and (3) homelessness complicates the treatment of many illnesses. Of course, certain diseases and treatments cut across these patterns and may occur in all three categories [41]. The complexity of identifying cause and effect in relation to health and homelessness suggests potential for analysis of SRH to contribute to improved understanding of homeless people’s experience.

### Drug use and health

The use of alcohol and other drugs are associated with a higher use of emergency departments [42, 43] and a higher likelihood of required hospitalization [44]. Drug abuse has a high number of negative health consequences. For example, alcohol use is associated with pancreatic diseases, hypertension and breast cancer [45]. This relation between alcohol and use of drugs with health problems is known to apply to the general population, but there has been a need for more research on the relationship with SRH in the Spanish homeless population. Alcohol use is one of the most important factors associated with the experience of acute homelessness [46, 47] and a causal factor in many health conditions [48]. Excessive alcohol use can also be associated with unstable housing [49] as well as higher emergency department use [50], suggesting that alcohol consumption is a key driver of emergency department use among homeless alcohol-dependent adults. Both homelessness and alcohol use influence the quality and quantity of interactions with health care providers [11] and alcohol use independently influences medical and psychiatric morbidity [51]. Alcohol use among the homeless may increase the burden of medical and psychiatric conditions, contribute to noncompliance with treatment interventions, and increase all-cause mortality [52]. From this evidence base, we can develop the research question: does *alcohol use* and *ever using drugs* have a significant association with SRH in Spanish homeless people?

### Health variables

As evidenced above, homeless people usually have worse physical and mental health than those who are housed [53], and they are exposed to more risk factors than the general population. The higher prevalence of cognitive and psychiatric disabilities in homeless people in comparison with the general population is also well established [54]. At the same time, homeless people have higher rates of mortality, injuries, suicide attempts and higher rates of infectious diseases [8].

In a study conducted by Chang et al. (2015) [55] with homeless adults, it was found that poor sleeping experience was also associated with poor/fair self-rated health, and that the topic merited further exploration. Furthermore, in Spain, the only health care available to people without a health card is the emergency department, and so this paper analyses the variables related to hospital or medical assistance and their relationship with SRH.

Given the lack of evidence of the relationship between these factors and SRH in the Spanish homeless population, we derive the question, do some *health variables* have a significant association with SRH among homeless people in Spain?

### Sociodemographic characteristics

Gender differences in health are well documented. Male mortality rates are higher than for females, although women report more symptoms, use more health care services than men, and tend to report worse SRH than men [56, 57]. However, this affirmation requires some clarification. For example, in a study conducted by Pinillos-Franco and García-Prieto (2017) [58] which focused on the working population in Spain (14,120 subjects aged between 25 and 65, extracted from European Union statistics), it was found that there was a gap in self-rated health but only for the less educated. The authors argued that women’s health is poorer among the less educated, mainly due to labour-market precarity and household conditions. In another study, Zajacova, Huzunbazar, and Todd (2017) [59] also found that women tended to register poorer SRH, compared to men, though less so among older age groups. Indeed, several studies suggested that women’s health ratings may be worse than men’s during earlier stages of adulthood [60] but this gender difference may attenuate or even disappear at older ages [61].

To sum up, evidence indicates that men and women evaluate their health differently [62] and SRH appears to predict mortality better for men than women [35, 37]. However, Zajacova et al. (2017) [59] found similarity among men and women’s concurrent SRH validity, for more comparable health factors.

Likewise, age is also associated with SRH, with older people more likely to report poorer SRH [58, 59], as health problems increase with age. The final emerging research question then is, do core *sociodemographic variables* have a significant association with SRH in the Spanish homeless population?

Building on consensus from previous studies [53, 63], it is important to analyse the factors that could be associated with poor health among homeless people in Spain and the resultant implications for health care provision. Therefore, the main purpose of the analysis which follows is to examine a range of factors which may be significantly associated with SRH of homeless people in Spain: *drugs use* (alcohol consumption, ever having used drugs); *health variables* (going to hospital once in the last year, going to the doctor in the last month, having a health card, having sleeping difficulties, having a disabling impairment); and *sociodemographic characteristics* (sex, age). Furthermore, as the only healthcare available to people in Spain who have no health card would be the emergency department, this paper analyses the variables related to hospital or emergency medical assistance and their relationship with SRH. The Spanish national survey of homeless people presents a robust data set for this analysis. This is the first national study of the homeless population in Spain that examines these variables.

## Method

### Secondary analysis of the Spanish national survey of homeless people

Our analysis of SRH and homelessness utilised data collected for the most recent Spanish Survey of Homeless People, which is a cross-sectional study of a representative national sample of the homeless population. This survey was funded and carried out by the Ministry of Economy, Industry and Competitiveness and administered by the National Institute of Statistics, within the scope of the Spanish Statistical Office. This National Institute allows researchers to work with the national open data file in order to undertake complementary secondary analysis of this valuable data set. The total sample interviewed for the Spanish Survey of Homeless People, was 3433 homelessness (*M*_*age =*_ 41.30, *SD* = 13.86). The authors of this paper removed 996 participants, who had missing data for at least one of the variables analysed, leaving a final sample of 2437 homeless people, analysed by a multinomial logistic regression modelling.

The period of survey recruitment was from 13 February until 25 march of 2012. The territorial scope determined included the municipalities with more than 20,000 people in the 50 provinces and the two autonomous cities of Spain. The information collection method was the Computer-Assisted Personal Interview (CAPI). All data were recorded directly onto a computer during the interviews with homeless people, conducted by 73 interviewers and 23 inspectors sensitized to this socially excluded population. The interviews covered sociodemographic characteristics, use of services and living conditions of homeless people. The authors then selected those questions that were associated with the aim of this study for analysis (see instrument paragraph below).

For the main survey, the lack of a simple framework for the direct selection of the target population made it necessary to use indirect sampling. The basic sampling units were accommodation and catering services provided specifically for the homeless population. The demand for these services in the previous year was used to estimate the total number of persons included as homeless in Spain. Homeless persons were defined as, aged 18 years old or over, who use centres that offer accommodation and/or catering services, located in municipalities with more than 20,000 inhabitants. This sampling was pre-determined by the Spanish National Institute of Statistics for the 2010 data collection [64]. This involved stratifying the accommodation centres and services in the survey and then devising a strategy for sampling individual homeless services users, with accompanying instructions for those conducting the interviews [64]. The data analysed in this paper were therefore collected in line with the official sampling strategy for the Spanish Survey of Homeless Persons.

The entire protocol and instrumentation were approved by the Committee of Good Practices of the European Statistical System (ETUCE), under the Directive 95/46/Parliament and the European Council of 24 October 1995 on the protection of individuals with regard to the processing of personal data and on the free movement of such data. The primary data collection was carried out over the course of six weeks and all participants consented to the study and data publication.

### Instruments

The variables used in this analysis are described below:

#### Alcohol and drugs use

In order to analyse the alcohol consumption the following question was used: *Could you tell me how often and what type of alcoholic drinks do you usually consume?* The Spanish National Institute for Statistics classified the responses into four categories considering the amount of pure alcohol consumed in a week: (1) Light, from 1 to 175 c.c. of pure alcohol/week; (2) Moderate, from 176 to 525 c.c. of pure alcohol/week; (3) High, from 526 to 700 c.c of pure alcohol/week; (4) Excessive, over 700 c.c. of pure alcohol/week. For this analysis, the authors of this paper used these responses to categorise participants according to their levels of alcohol risk: abstainers (non-drinkers), moderate risk drinkers (light and moderate), high-risk drinkers (high and excessive). With respect to drugs use, *homeless* people interviewed were asked the question: *Have you ever used drugs?* Types of drugs included marijuana or hashish, cocaine, heroin, and other substances. The possible responses were *yes* or *no*. Other survey information which did not relate directly to the objectives of this paper, has not been considered here.

#### Health variables

The following variables were selected (coded as yes or no): attended *hospital at least once in the last year*; attended *the doctor in the last month*; *have a health card*; have *sleeping difficulties*; *have a disabling impairment* (coded as physical, sensory, intellectual, psychiatric, or none).

#### Self-rated health (SRH)

SRH was derived from a single question in the Spanish survey: Currently, how is your state of health? The five possible responses (very good, good, regular, poor and very poor) were categorized by the authors in *good* (very good and good health), *regular* and *poor health* (poor and very poor self-rated health).

### Statistical analyses

The relationships between *drug use*, *health variables* and *SRH* were examined using multinomial logistic regression modelling. *Health perception* was modelled as the dependent variable, with “poor health” forming the reference category. Medical variables were modelled as independent variables or factors (“no” was the reference category for each variable and “none” was the reference category to the variable *have a disability*). Sociodemographic factors and drugs use were also modelled as factors. The analysis was performed with the statistical package SPSS version 21.0 for PC. All reference categories are shown on Table 2.

## Results

The sample consisted of 2437 adults aged 18 years and over (Table 1). Just over four fifths of respondents were male, with less than a fifth female. The age profile of respondents was more evenly spread, although slightly skewed to the older (over 40 years) age groups. A majority of applicants reported Good or Regular health, but a significant minority (almost two fifths) reported Poor health. A slight majority of respondents reported abstaining from alcohol (54.5%) and never having used drugs (55.6%). The vast majority of respondents reported having a health card (85.4%), but only just over half (56.3%) had visited the doctor in the last month and less than a quarter (23.9%) had been in hospital at least once in the last year. Just less than one fifth reported having an impairment leading to disability – mainly physical impairments but with small proportions reporting sensory/intellectual or psychiatric conditions. More than two fifths (44.4%) of homeless people in the Spanish national survey reported having difficulties sleeping. Overall then, there was some variation in the characteristics and health indicators across the Spanish homeless population. Multinomial logistic regression modelling was carried out to further interrogate the association between SRH and the other variables.

**Table 1:** Demographic characteristics of the current sample

Factors	Categories	*n*	*%*
SRH	Good	403	16.5%
	Regular	1067	43.8%
	Poor	967	39.7%
Alcohol consumption	Abstainer^a^	1327	54.5%
	Moderate Risk^b^	1039	42.6%
	High-Risk^c^	71	2.9%
Sex	Male	2043	83.8%
	Female	394	16.2%
Ever use drugs	Yes	1082	44.4%
	No	1355	55.6%
Hospital once last year	Yes	583	23.9%
	No	1854	76.1%
Go to the doctor last month	Yes	1372	56.3%
	No	1065	43.7%
Health card	Yes	2080	85.4%
	No	357	14.6%
Sleeping difficulties	Yes	1081	44.4%
	No	1356	55.6%
Disability	Physical	267	11.0%
	Sensory or intellectual	32	1.3%
	Psychiatric	115	4.7%
	None	2023	83.0%
Age	18–29	421	17.3%
	30–39	496	20.4%
	40–49	745	30.6%
	50 or more years	775	31.8%
Total		2437	100.0%

^a^Not having consumed alcohol in a week; ^b^Between 1 and 525 cc of alcohol per week; ^c^Drinking more than 526 cc per week

The multinomial logistic regression modelling analyses the SRH perception as a dependent variable and its relationship with the factors mentioned above. The likelihood ratio test of the final model against the ones where all the parameter coefficients are 0 (null), conclude that the final model is outperforming the null (χ^2^ = 552,220; *df* = 28; *p* < .001). The corrected percentage of the classification table, between the observed and predicted data, according to the model, is 55.9% of global success.

### Drugs and sociodemographic variables

The results (Table 2) showed that the majority of individuals who were *abstainers* were significantly more likely than *higher-risk drinkers* to perceive good health (OR = 2.66, 95% CI [1.07, 6.62]) and more likely to perceive regular health (OR = 2.38, 95% CI [1.34, 4.22]) than poor health. The substantial minority of individuals who *ever used drugs* were less likely, than the majority who *never used drugs*, to perceive good health (OR = 0.69, 95% CI [0.53, 0.91]) than poor health. *Males* were significantly more likely than females to perceive good health (OR = 2.00, 95% CI [1.37, 2.93]) and regular health (OR = 1.66, 95% CI [1.28, 2.151]) than poor health. The *oldest* homeless people were more likely to perceive poor health than the younger age groups (see Table 2), and notably the *youngest* people (18–29 years old) were more likely than the *oldest* (> 50 years old) (OR = 7.21, 95% CI [4.86, 1.68]) to perceive good health and regular health (OR = 2.72, 95% CI [1.99, 3.73]) than poor health.

**Table 2:** Results of the logistic regression model examining the factors in health perception

	Good health versus Poor health	*p*	Regular health vs Poor health	*P*
Sex				
Male	2.00* [1.37, 2.93]	< 0.001	1.66* [1.28, 2.15]	< 0.001
Female	Reference		Reference	
Alcohol consumption				
Abstainer^a^	2.66* [1.07,6.62]	0.035	2.38* [1.34, 4.22]	0.003
Moderate-Risk^b^	2.20 [0.88,5.47]	0.090	2.05* [1.15, 3.64]	0.014
High-Risk^c^	Reference		Reference	
Ever use drugs^a^	0.69* [0.53, 0.91]	0.008	0.90 [0.74, 1.09]	0.306
Hospital once last year ^a^	0.49* [0.35, 0.68]	< 0.001	0.52* [0.42, 0.65]	< 0.001
Go to the doctor last month^a^	0.60* [0.46, 0.78]	< 0.001	0.59* [0.48, 0.72]	< 0.001
Health card^a^	1.66* [1.15, 2.40]	0.006	1.32 [0.99, 1.75]	0.053
Sleeping difficulties ^a^	0.29* [0.22, 0.38]	< 0.001	0.43* [0.35, 0.52]	< 0.001
Disability				
Physical	0.157* [0.07, 0.31]	< 0.001	0.412* [0.30, 0.56]	< 0.001
Sensory or intelectual	0.378 [0.08, 1.76]	0.215	0.91 [0.41, 1.99]	0.815
Psychiatric	0.294* [0.12, 0.71]	0.007	1.23 [0.80, 1.88]	0.343
No one	Reference		Reference	
Age				
18–29	7.21*[4.86, 10.68]	< 0.001	2.72* [1.99, 3.73]	< 0.001
30–39	3.18* [2.17, 4.64]	< 0.001	2.05* [1.56, 2.70]	< 0.001
40–49	1.86* [1.31, 2.66]	0.001	1.44* [1.14, 1.82]	0.002
≥ 50	Reference		Reference	

^a^Bad health is the reference category. Results are reported as odds ratios with 95% confidence intervals shown in parentheses *p < 0.05. ^a^Reference category is ‘no’

### Health variables

Several health factors were associated with health perception in homeless people. Those who were *in hospital last year* for *at least one nig*ht, were significantly less likely, than those who never went to hospital to perceive themselves as in good health (OR = 0.49, 95% CI [0.34, 0.68]) and regular health (OR = 0.52, 95% CI [0.52, 0.65]) than poor health. These results show a similar pattern as for the variables attended *the doctor in the last month*, *sleeping difficulties* and *have a disability/impairment -* all of which were associated with lower likelihood of perceiving good health or regular health than poor health. Furthermore, homeless people who had a *health card* were significantly more likely (OR = 1.66, 95% CI [1.15, 2.40]) to perceive good health than poor health.

## Discussion

The main purpose of this study was to examine whether drug use, health variables and sociodemographic characteristics were significantly associated with SRH in the Spanish homeless population. The results showed that to be *male*, an *abstainer*, have a *health card* and to be in the *youngest age groups* were significantly associated with perceiving good health amongst homeless people in Spain. On the other hand, the other variables analysed were all significant risk factors associated with poor SRH: having *ever used drugs*, *spent a night in hospital once in the last year*, *gone to the doctor in the last month*, having *sleeping difficulties*, *disability* and *older* age.

These results are consistent with expectations and other international studies of homeless people, where the use of drugs and alcohol is associated with illness or poor health, increased probability of mortality and continuing homelessness [65]**.** The recent evidence from Spain suggests little significant change from Australian findings from 1990, which established that alcohol was associated with ill health in the Australian homeless population [66]. Similarly, substance use and other health issues continue to emerge as problems experienced by Spanish homeless people [46, 67]. It is possible that the use of drugs and alcohol is a coping strategy for homeless people, making it difficult to address drug consumption, despite the direct affect on their health [68].

To have a *health card* appears a clear prevention factor. This concurs with findings from other studies which show that not to have a health card prevented homeless people from accessing required health care services [4, 69]. Homeless persons common experience of encountering barriers to obtaining primary health care, helps explain their relatively poorer health [4, 10]. While a high proportion (85.4%) of Spanish homeless people did have a health card, ensuring this translates into access to health care remains an important issue. Moreover, in Spain, illegal immigrants who are not eligible to obtain a health card would have to use emergency departments for health care **(**Real Decreto-ley 16/2012). Results showed that *going to the doctor in the last month* or *going to the hospital at least one night last year* are also associated with poor health. Again, this is in agreement with other studies indicating that once homeless people have used emergency departments, they usually visit again and have inpatient readmissions after hospital discharge within the next month [70]. In addition, *sleeping difficultie*s [71] which were reported by more than two fifths of Spanish homeless people and *physical and psychiatric disabilities* are also associated with a poor SRH. Just less than a fifth of Spanish homeless people identified as disabled but international evidence suggests homeless people may experience disability at a younger age than the general population [63], and are also more likely to have an intellectual disability, possibly associated with the relationship between drug use and mental health problems [72].

In Spain, 61% of the homeless population is older than 40 years, with 30.8% older than 50 years. Our research shows that as age increases, the likelihood of reporting good health or regular health against poor health was reduced. While this could be a general finding in relation to the effects of age, research has demonstrated that older homeless people have higher rates of geriatric syndromes than in the general/housed older population [73]. In fact, homeless people begin to be admitted to hospital for medical conditions 10 or 15 years earlier than the general population [74]. Our results, in line with other studies of the Spanish population, showed how SRH deteriorates as age increases (11). It is, however, also important to consider the demonstrated bias within the general population older than 70 years old, who tend to report a better SRH than objective health [75]. Nevertheless, according to our results, this bias does not seem to happen within the Spanish homeless population, where the SRH is poorer as age increases, independently of the most advanced age stage. Regarding sex, our results are again in agreement with those for the general Spanish population, and with another study in the homeless population [28], in that females tend to have worse health than males [27] independently of their age [76]. In their international review, Wolf et al. (2016) [14] found a lack of clear evidence on the health of homeless women, compared to homeless men.

It is important to consider the policy implications of these research findings for future policy and practice. Henwood, Cabassa, Craig, and Padget, (2013) [9] highlight the benefits of ‘Permanent Supported Housing’ (PSH), as a solution to homelessness and key to improving health. Research evidence also indicates that interventions involving peers in supporting lifestyle and health behaviour changes can be effective in settled accommodation [9, 77]. Having a home may also have physical health benefits, resulting from decreased cortisol levels [78].

The acknowledgement of housing as a social determinant of health can inform wider health, care and housing policy, including service co-ordination. For example, health and care interventions should be sensitive to aging, and designed to achieve sustainable neighbourhoods which support positive health and well-being outcomes [9].

The health care needs of homeless people have also been addressed through the provision of medical services in the shelters and day services for homeless people. For example, the New York homeless shelter system provides primary care and mental health services on-site in some of the shelters in the form of clinics staffed by nurse practitioners, internists, and psychiatrics. Schanzer, Dominguez, Shrout, and Caton (2007) [12] conducted a longitudinal (18 month) study of 445 individuals from their entry into the homeless shelter system, finding that some aspects of the participants’ health status showed improvement. In the European context, Anderson and Ytrehus (2012) [10] concluded that while homeless people’s exclusion from mainstream health services sometimes necessitated direct health care provision in homelessness services, the more effective long term policy and practice goal would be to improve access to settled housing and national health services.

Finally, Pinillos-Franco and García-Prieto (2017) [58] highlighted the importance of promoting education and access to the labour market as a strategy to improve SRH which raises the general health level of the population and tends to reduce socioeconomic gender inequalities over time. Education and employability strategies could be integrated into housing-led solutions to homelessness alongside health interventions.

### Limitations

The study has some potential limitations. Although we have argued for the validity of SRH, the use of self-reporting for both general health, and the rest of the associated variables analysed, may be considered itself a limitation. Nevertheless, checklists of conditions have been shown to encourage reporting by the genuinely ill and to give an accurate reflection of health needs [79]. However, there is scope for future research to analyse and compare SRH with objective measures of health in the Spanish homeless population. There is also scope for developing a more sophisticated measurement of the variable for use of alcohol and other drugs.

We have used SRH to measure health perception so it is most important to consider the validity of the measure. A key issue is the reduction in predictive validity as age increases found by Zajacova and Woo (2016) [39], suggesting SRH is most useful for the younger age group participants. Furthermore, comparisons within older age groups should be treated with caution due to the possibility of variation due to differential reporting in older age. Allowing for these limitations, however the analysis contributes new evidence on SRH within the Spanish homeless population.

## Conclusions

Scientific reviews have increasingly emphasized the need for homelessness interventions to be sensitively designed to meet particular needs within the population [80]. Substance use [72, 81], illness and disabilities [63] have been demonstrated to be influential in a homeless situation. Similarly, our results showed that drug use and disabilities negatively affect the SRH of homeless people, potentially forming a cycle that can be difficult to interrupt. However, our statistical analysis indicates that interventions to reduce drug consumption and ensure access to a health card/health services, as well as enhancing services for older, female and disabled homeless people are all measures which could improve health and well-being for those who face homelessness.

It is important to consider age-specific interventions. Analysis of the Spanish data set indicated those aged 18–29 years were more likely to perceive good or regular health than those aged over 50 years. However, prior research also indicates that enduring adolescent homeless situations affect physical and mental health [82], while some young homeless people have some feelings of embarrassment and experiences of being denied care due to being under 18 years old [70]. A key contribution of this analysis appears to be that while there is a need for preventive interventions targeted at younger homeless people, the self-rated poor health of older age groups also requires attention. Further, given the possible limitation of reporting tendencies in older age groups, age specific interventions merit further investigation.

In conclusion, despite some limitations, this study presents new evidence on how substance use, medical and demographic variables are associated with SHR of Spanish homeless people. Improved understanding of the key factors that influence the self-perception of health in turn contributes to development and delivery of preventive interventions. Delivering more effective medical care is critical to improving the well-being of homeless people, as is enabling their access to suitable housing.

Despite the many challenges in tackling homelessness, emerging evidence informed strategies to address the related housing health and social consequences clearly need to include affordable and supportive settled housing, targeted harm-reduction and mental health strategies, inclusive approaches to individual and community empowerment, and poverty-reduction strategies [83, 84]. There is considerable scope to improve evidence-based practice in the health-care sector to ensure that excluded groups, such as the homeless receive the health care they need. However, Government leadership is also crucial to deliver on the research findings presented here, especially in times of economic hardship. Employment, housing, health and welfare policies are all fundamental to increasing the effectiveness of national homelessness strategies in resolving homelessness and enhancing homeless people’s self-rated health.

## Data Availability

The dataset used and/or analyzed during the current study is available from the corresponding author on reasonable request.

## Abbreviations

ETUCE: Committee of Good Practices of the European Statistical System
SRH: Self-Rated Health
